# The Association Between Social Determinants of Health (SDoH) and Mental Health Status in the US

**DOI:** 10.3390/ejihpe15050087

**Published:** 2025-05-17

**Authors:** Farhana Faruque, Gulzar H. Shah, Robert M. Bohler

**Affiliations:** Jiann-Ping Hsu College of Public Health, Georgia Southern University, Statesboro, GA 30460, USA; fz00290@georgiasouthern.edu (F.F.); rbohler@georgiasouthern.edu (R.M.B.)

**Keywords:** social determinants of health, mental health, income disparities, transportation barriers, healthcare access, job loss, education, mental health equity

## Abstract

Social determinants of health (SDoH) are considered significant determinants of mental health. This study examines the association between SDoH and mental health status in the United States. We analyzed 2023 Behavioral Risk Factor Surveillance System (BRFSS) data from 183,318 U.S. adults using multinomial logistic regression. Several SDoH were significantly linked to the frequency of poor mental health days. After adjusting for all covariates, individuals facing difficulty paying utility bills had lower odds of experiencing episodic (vs. chronic) poor mental health (AOR = 0.47, *p* = 0.031). Transportation challenges were associated with lower odds of episodic distress rather than chronic mental health issues (AOR = 0.35, *p* = 0.026). Individuals who were unable to afford a doctor or who experienced employment loss had significantly lower odds of reporting no poor mental health days compared to reporting chronic poor mental health, with adjusted odds ratios of 0.37 and 0.84, respectively. Non-Hispanic Whites and males were more likely to report chronic poor mental health. Policies that prioritize economic stability and job security, reliable transportation, and equal access to education and healthcare are crucial for promoting mental health equity across diverse populations.

## 1. Introduction

Mental health disorders have increasingly been recognized as a significant public health issue in the United States (U.S.). According to the Centers for Disease Control and Prevention, millions of adults are affected by depression, anxiety, and stress each year, and rates of depression and anxiety have been increasing over time (Terlizzi & Zablotsky, 2024). Approximately 52.9 million, or 23.1% of adults in the U.S., experienced mental illness in 2023 (National Institute of Mental Health, 2023). Recent data have shown that 14.1% of people in the U.S. reported poor mental health for 14 days or more within 30 days (CDC, 2021). Like the situation in the U.S., depression and anxiety are global challenges, with a recent global uptick in their prevalence of 25% in the aftermath of the COVID-19 pandemic (Santomauro et al., 2021). Poor mental health not only impacts the personal well-being of individuals but also burdens families, communities, and social structures due to its negative impact on productivity, harmony (e.g., through crimes), and healthcare resources (Highland et al., 2020).

Poor mental health refers to emotional states (such as persistent sadness, anxiety, or stress) that interfere with an individual’s ability to function in daily life. According to the Centers for Disease Control and Prevention (CDC), “poor mental health” is defined as the number of days during the past 30 days when an individual’s mental health was “not good”, encompassing experiences related to emotional distress, depression, and stress (CDC, 2024).

The COVID-19 pandemic highlighted the crucial role of social determinants of health (SDoH) in shaping mental health outcomes (Breslau et al., 2023). The World Health Organization describes SDoH as “the non-medical factors that affect health outcomes. These include the conditions people experience throughout their lives—where they are born, grow, live, work, and age—as well as the broader social, economic, and systemic factors that shape their daily lives (WHO, 2025). Several studies found that anxiety, depression, and psychological distress increased during the pandemic due to the persisting inequitable access to SDoH, especially among groups with certain socioeconomic and race-related vulnerabilities (Melchior et al., 2024; G. H. Shah et al., 2020). This crisis exacerbated existing health disparities and inequities and reinforced the need to address SDoH in mental health policies (A. D. Shah et al., 2024).

Moreover, vulnerabilities are not uniformly distributed (Alghamdi et al., 2023). Certain groups—such as immigrants, racial and ethnic minorities, people with low income, and those who have experienced trauma—often experience disproportionate mental health challenges (Garcini et al., 2022). Immigrants may face chronic stress from language difficulties, discrimination, and uncertain legal status (Held et al., 2025). Minority populations often encounter systemic inequities in education, healthcare, and employment that contribute to psychological distress (Cotton & Shim, 2022). Individuals with a history of trauma, such as violence or displacement, may not have the support they need to recover (Mao & Agyapong, 2021). These combined challenges underscore the need to examine how SDoH affect mental health across diverse demographic groups (Funer, 2023).

While individual-level determinants of mental health challenges are well-researched, broader societal and environmental factors remain understudied. Given an increasing consensus among public health practitioners for addressing upstream factors for downstream elimination of disparities, the reasons for mental health disparities are now increasingly being traced to inequities in SDoH (Kalu et al., 2024a, 2024b). Social determinants such as income, education, employment, race/ethnicity, and community environments are ever more recognized as the root causes of overall health status, including mental well-being (Alegría et al., 2023; Kalu et al., 2024b). Economic stability, access to quality healthcare services, and superior neighborhood safety can play a critical role in alleviating mental health problems (Weida et al., 2020; Williams & Cooper, 2019).

Social determinants of health connect to mental health status in several ways. First, inequities in SDoH, like economic instability, homelessness, food insecurity, and poor education, can lead to chronic stress as well as post-traumatic stress (Kalu et al., 2024a; Mao & Agyapong, 2021). In turn, aggravated chronic stress raises the risk of anxiety and depression (Remes et al., 2021; Guan et al., 2022). Secondly, SDoH greatly influence access to essential resources such as education and healthcare. Limited access to critical resources hinders early intervention, worsening mental health over time (Wilkinson et al., 2023). Finally, social inequalities and inequities, such as lack of community support and discrimination against minorities, exacerbate feelings of helplessness and loneliness, adversely influencing mental health (Keim-Klärner et al., 2023; Pearce et al., 2023). Furthermore, disparities in healthcare access among underprivileged populations not only deepen mental health inequities but also increase the burden of chronic diseases such as diabetes, driven by socioeconomic and lifestyle factors that shape health outcomes (Park & Berkowitz, 2025; Zerin et al., 2021).

Research has also shown the intersectionality of inequities surrounding SDoH, such as food insecurity, higher healthcare costs, and worsening mental health (Park & Berkowitz, 2025). Similarly, the combined influence of poor education and income levels negatively impacts mental health and its treatment outcomes (Lee & Allen, 2023). Given the intersectionality of various deficits in SDoH and their combined negative health impacts, understanding the relationship between SDoH and mental health outcomes is pivotal (Alghamdi et al., 2023; Funer, 2023). Lee and Allen (2023) found a correlation between the risk of depression and income inequity. However, additional research is needed to elucidate how different social factors influence psychological health through diverse mechanisms (Kammer-Kerwick et al., 2024). Moreover, prior research suggests that integrating social services and mental health care is crucial to an individual’s overall well-being and efficient service delivery (Cochran et al., 2022; Crocker et al., 2020). Numerous studies focus on factors such as education or income without considering their combined effect, thereby overlooking the socio-structural influences (Alegría et al., 2023; Tan, 2024; Makhlouf & Lalley, 2023).

The current study examined the influence of multiple SDoH in adults, including education, income, job status, insurance coverage, housing stability, and access to healthcare, on mental illness in the U.S. In general, limited or inequitable access to SDoH is expected to be associated with a higher number of reported days of poor mental health. Findings from our study will inform tailored interventions and policies targeted to mitigate the root causes of disparities in mental health.

## 2. Materials and Methods

### 2.1. Data Source

This quantitative cross-sectional study used secondary data from the 2023 Behavioral Risk Factor Surveillance System (BRFSS) Survey. Conducted annually by the Centers for Disease Control and Prevention (CDC), the BRFSS is a state-based, nationally representative survey designed to collect data on health-related risk behaviors, chronic health conditions, and the use of preventive services. The 2023 BRFSS incorporated a complex sampling design, using Disproportionate Stratified Sampling (DSS) for landline data collection and Random Digit Dialing (RDD) for cell phone interviews. This methodology ensures adequate representation across the United States’ diverse demographic groups and geographic regions.

### 2.2. Data Collection

Data were collected between January and December 2023 via telephone interviews, incorporating landline and mobile samples. Interviews were administered in English and Spanish to ensure inclusivity. Respondents were selected through stratified random sampling, targeting non-institutionalized U.S. adults. Each selected household was contacted multiple times to encourage participation. A structured questionnaire encompassed items related to health status, social determinants of health (SDoH), and demographic characteristics. Survey responses with missing data for key variables, such as mental health status, were excluded from this study’s analysis.

### 2.3. Variables

#### 2.3.1. Dependent Variable

The dependent variable for this study was mental health status, operationalized as the number of poor mental health days reported by study participants in the past 30 days. The responses were collected at three levels: no poor mental health (0 days), episodic poor mental health (1–13 days), and chronic poor mental health (14–30 days). This classification captures the mental health burden experienced by individuals, ranging from no distress to chronic distress. This stratification is used by the Centers for Disease Control and Prevention’s Behavioral Risk Factor Surveillance System (BRFSS), which utilizes these categories to assess mental health status (CDC, 2024).

#### 2.3.2. Independent Variables

The independent variables included a range of SDoH, classified under the following categories: economic stability, access to and quality of education, social and community context, neighborhood and built environment, and access to and quality of healthcare. Economic stability, represented by annual household income, was categorized into <USD 15,000, USD 15,000–24,999, USD 25,000–49,999, USD 50,000–74,999, and ≥USD 75,000. Employment loss or reduction was determined by whether respondents reported losing employment or having their working hours reduced in the past 12 months (yes/no). Educational attainment, a measure of access to and quality of education, was categorized as less than high school, high school graduate, some college, or college graduate. The social and community context included marital status, categorized as married, divorced, widowed, separated, or never married. Food assistance participation was operationalized through the reported frequency of receiving food stamps, also known as “Supplemental Nutrition Assistance Program (SNAP)”, 12 months before the survey (never, rarely, sometimes, or often). Neighborhood and built environment variables included housing instability, measured by whether respondents had trouble paying their mortgage, rent, or utility bills in the past 12 months (yes/no), and transportation barriers, which assessed the lack of reliable transportation for essential activities in the past 12 months (yes/no). Healthcare access and quality variables included health insurance coverage (insured/uninsured), affordability, assessed by whether respondents could afford to see a doctor in the past 12 months (yes/no), and time since the last routine checkup, categorized as within the past year, 1–2 years ago, or more than 2 years ago.

The demographic variables included age (grouped into 18–24, 25–34, 35–44, 45–54, 55–64, and ≥65 years), gender (male/female), and race/ethnicity, which was categorized as non-Hispanic White, non-Hispanic Black, Hispanic, Asian, or other. Each variable was systematically coded and categorized to ensure consistency and reliability in the analysis.

### 2.4. Data Analysis

Descriptive statistics were computed to summarize the distribution of demographic characteristics, SDoH variables, and mental health status across the study population. Weighted frequencies and percentages were used to account for the complex survey design. For multivariable analysis, we initially considered ordinal logistic regression and tested the assumption of proportional odds, which our model violated. Multinomial logistic regression analysis was eventually employed to assess the association between SDoH indicators and mental health status, comprising three categories. Adjusted odds ratios (AORs) and 95% confidence intervals (CIs) were calculated to estimate the strength of associations, controlling for potential confounders such as age, gender, and race/ethnicity. Our analyses utilized the statistical weights provided in the BRFSS dataset to account for the complex survey design, including oversampling of specific populations and nonresponse adjustments, thereby enhancing the generalizability of our findings to the U.S. adult population aged 18 years and older. All analyses were conducted using IBM SPSS Statistics Software, version 29.0.2.0 (2023).

## 3. Results

### 3.1. Descriptive Characteristics of the Adult Respondents

**Figure 1:**
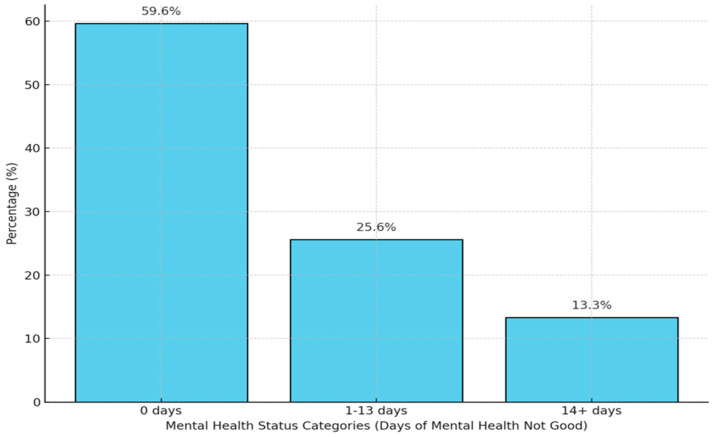
Percent distribution of mental health status based on days of poor mental health from the 2023 BRFSS Survey.

Figure 1 depicts the composition of study participants by the number of poor mental health days in the 30 days prior to participation in the survey. Although 59.6% had no poor mental health (0 days of poor mental health), one in four (25.6%) had episodic poor mental health with 1 to 13 days in 30 days. A small but notable proportion, 13.3%, had chronic poor mental health—14 to 30 days per month. Findings indicate that 6.8% (n = 12,500) of participants reported experiencing transportation barriers, while 93.2% (n = 170,818) did not (Table 1).

### 3.2. SDoH Associated with Mental Health

The multinomial logistic regression analysis results show significant associations between many social determinants and mental health status (Table 2). Gender demonstrated a significant relationship with mental health. Males, compared to females, had significantly lower odds of experiencing episodic (1–13 days) rather than chronic (14+ days) poor mental health (AOR = 0.96, 95% CI = 0.94–0.99, *p* = 0.040). Additionally, males had significantly lower odds of reporting no poor mental health days compared to chronic poor mental health (AOR = 0.92, 95% CI = 0.89–0.95, *p* < 0.001) in the past 30 days. Race and ethnicity showed significant variations in the odds of poor mental health outcomes. Compared to Hispanic individuals, Non-Hispanic Whites were less likely to report episodic poor mental health (AOR = 0.73, 95% CI = 0.58–0.92, *p* = 0.008) and no poor mental health days (AOR = 0.73, 95% CI = 0.58–0.92, *p* = 0.004) rather than chronic poor mental health. Educational level showed a protective effect against poor mental health days. Compared to individuals with at least four years of college education, those with some college education had lower odds (AOR = 0.57, 95% CI = 0.34–0.96, *p* = 0.033) of experiencing episodic rather than chronic poor mental health, suggesting a significant protective effect. Similarly, high school graduates had lower odds (AOR = 0.59, 95% CI = 0.35–1.00, *p* = 0.05) of experiencing episodic poor mental health compared to chronic poor mental health.

People who “usually” received SNAP benefits were more likely to have episodic mental health issues (1–13 days a month) rather than frequent or chronic issues (14–30 days), compared to those who never received SNAP (AOR = 1.75, 95% CI: 1.23–2.51, *p* = 0.002). Similarly, those who “always” received food assistance were more likely to experience occasional rather than chronic poor mental health (AOR = 1.70, 95% CI: 1.20–2.40, *p* = 0.003). However, food assistance did not make a significant difference in the chances of having no poor mental health days compared to having chronic mental health problems. This suggests that while SNAP participation negatively impacts mental health, its effects may fluctuate over time rather than remain consistently severe.

In addition, individuals who struggled to pay utility bills had significantly lower odds of experiencing episodic rather than chronic poor mental health (AOR = 0.47, 95% CI = 0.19–0.97, *p* = 0.031). They also had lower odds of having no poor mental health compared to experiencing chronic poor mental health (AOR = 0.24, 95% CI = 0.09–0.6, *p* = 0.003).

Annual household income demonstrated a significant association with mental health. Compared to individuals earning USD 200,000 or more, those with incomes of USD 100,000 to USD 199,000 had higher odds of experiencing episodic (1–13 days) rather than chronic (14+ days) poor mental health (AOR = 1.36, 95% CI = 1.27–1.45, *p* < 0.001), as did individuals earning USD 50,000 to USD 100,000 (AOR = 1.46, 95% CI = 1.46–1.54, *p* < 0.001). Similarly, those earning USD 35,000 to < USD 49,000 (AOR = 1.33, 95% CI = 1.33–1.40, *p* < 0.001), USD 25,000 to USD 34,000 (AOR = 1.26, 95% CI = 1.26–1.34, *p* < 0.001), and USD 15,000 to USD 24,000 (AOR = 1.17, 95% CI = 1.17–1.27, *p* < 0.001) were more likely to report episodic rather than chronic poor mental health. However, while comparing no poor mental health vs. chronic poor mental health days, Individuals earning USD 100,000 to USD 199,000 (AOR = 0.45, 95% CI = 0.34–0.60, *p* < 0.001), USD 50,000 to USD 100,000 (AOR = 0.57, 95% CI = 0.48–0.69, *p* < 0.001), USD 35,000 to < USD 50,000 (AOR = 0.78, 95% CI = 0.67–0.91, *p* = 0.002), and USD 25,000 to USD 34,000 (AOR = 0.81, 95% CI = 0.69–0.95, *p* = 0.011) had lower odds of reporting no poor mental health days. This means that individuals in lower income brackets are more likely to experience chronic poor mental health (14+ days) rather than having no poor mental health (0 days). This suggests that annual household income has a negative impact on mental health, whether in the form of episodic or chronic poor mental health.

Individuals facing transportation barriers had significantly lower odds of reporting episodic rather than chronic poor mental health (AOR = 0.35, 95% CI = 0.14–0.88, *p* = 0.026), suggesting a higher likelihood of experiencing chronic poor mental health problems rather than episodic or temporary issues. Additionally, those with transportation barriers had reduced odds (AOR = 0.21, 95% 95% CI: 0.07–0.58, *p* = 0.003) of reporting no days of poor mental health compared to chronic poor mental health. This finding suggests that they are more likely to experience chronic or persistent mental health problems than those who have no mental health issues.

Individuals who could not afford to see a doctor had lower odds of reporting episodic rather than chronic poor mental health (AOR = 0.59, 95% 95% CI: 0.29–1.195, *p* = 0.142), suggesting a trend toward a higher likelihood of experiencing chronic poor mental health rather than episodic issues, though this finding was not statistically significant. Additionally, those unable to afford a doctor had significantly reduced odds (AOR = 0.37, 95%CI: 0.21–0.65, *p* = 0.004) of reporting no days of poor mental health compared to chronic poor mental health. This finding suggests that financial barriers to healthcare are associated with a greater likelihood of experiencing chronic or persistent mental health problems rather than having no mental health issues.

Individuals who experienced employment loss or reduction had no significant difference in the odds of reporting episodic rather than chronic poor mental health (AOR = 1.17, 95% CI: 0.90–1.51, *p* = 0.236). This suggests that employment instability alone may not be a primary factor distinguishing between short-term and long-term mental health distress. However, those who faced employment loss had significantly lower odds (AOR = 0.84, 95% CI: 0.77–0.91, *p* < 0.001) of reporting no poor mental health compared to chronic poor mental health. This finding highlights that employment instability is closely linked to an increased risk of persistent mental health distress rather than an absence of mental distress.

Overall, the majority of results support the expectation that the odds of poor mental health among U.S. adults are raised by limited or inequitable access to SDoH, including lower income, limited education, transportation barriers, and lack of healthcare access.

## 4. Discussion

This study’s purpose was to provide a deeper insight into how multiple social determinants of health were associated with mental health outcomes by integrating and expanding on prior research. Analysis of a nationally representative dataset, the 2023 BRFSS, underscored the significant impact of several demographic, socioeconomic, and environmental factors in shaping mental health disparities, like a previous study (Nelson, 2022) which analyzed 2017–2019 BRFSS data to assess the relationship between SDoH burden and overall health (days of poor physical health, and poor mental health). Our study added additional insight using a range of social determinants of health (SDoH), employed a more rigorous statistical technique, and took a more nuanced approach by examining the association of SDoH with episodic or chronic poor mental health days.

Our results show that men are more likely to report no poor mental health days at all, but when they do, they tend to experience chronic rather than episodic poor mental health. Specifically, men had lower odds of reporting episodic poor mental health (1 to 13 days) compared to chronic poor mental health (14 or more days of the month). Our study also showed that men were significantly more likely to have no reported days of poor mental health days rather than chronic poor mental health. Thus, our results support that men were more likely to either experience no mental health distress at all or chronic distress. In contrast, women were more likely to experience episodic rather than chronic mental health distress. These findings align with those of Baños and Miragall (2024), who suggested that men may under-report mental health issues due to societal norms discouraging emotional expression (Baños & Miragall, 2024). Similarly, Wilkinson et al. (2023) found that women faced higher rates of depressive disorders, stimulated by overlapping challenges such as caregiving responsibilities and financial disparities, and Terlizzi and Zablotsky (2024) found that, in general, women reported depression and anxiety symptoms more often than men.

Notable ethnic and racial disparities in mental health outcomes were observed in our study. The research noted that compared to Hispanic individuals, non-Hispanic whites were more likely to report chronic poor mental health, rather than no mental health issues or episodic mental health issues. This finding contrasts with prior research by McGregor et al. (2020), who highlighted racial disparities in access to mental health treatment (McGregor et al., 2020), but aligns with the research of Terlizzi and Zablotsky (2024), who found that non-Hispanic whites were more likely to report depression and anxiety symptoms compared to Hispanics. While Garcini et al. (2022) emphasized that Hispanic populations face unique mental health challenges, including discrimination and immigration-related barriers, our results suggest that despite these challenges, chronic poor mental health is more prevalent among non-Hispanic Whites (Garcini et al., 2022).

This study found that educational level functioned as a protective factor against poor mental health outcomes. Individuals with higher education levels had significantly lower odds of experiencing episodic or chronic poor mental health compared to those with lower education levels. These findings align with prior research indicating that higher education provides greater access to resources, better job prospects, and improved mental well-being (Wilkinson et al., 2023). Additionally, Lee and Allen (2023) suggest that education mitigates the negative effects of income inequality on mental health, an aspect that should be further explored in future studies (Lee & Allen, 2023).

Our study found that participation in food assistance programs, such as SNAP, was linked to mental health outcomes. Individuals who consistently received food assistance (“usually” or “always”) were more likely to experience episodic rather than chronic poor mental health. This suggests that food support may lower short-term distress, consequently protecting against chronic mental health struggles. This finding aligns with Ettinger de Cuba et al. (2019), who noted that while SNAP can reduce financial stress, it often does not fully eliminate mental health challenges (Ettinger de Cuba et al., 2019). Similarly, Weida et al. (2020) showed that food insecurity increases anxiety and depression, whereas improving food access offers some relief (Wolfson et al., 2021). Research evidence also indicates that the mental health benefits of food assistance programs vary depending on their consistency, adequacy, and accessibility (Dean et al., 2020). These results highlight the urgent need for broader policies that address food access alongside other social determinants (Leddy et al., 2020). Research shows that food insecurity contributes to chronic psychological distress by creating ongoing uncertainty about access to basic needs, which intensifies stress and emotional vulnerability over time (Wolfson et al., 2021; Dean et al., 2020). In addition, studies have found that food insecurity is often accompanied by social isolation and loneliness, which independently increase the risk of depression and anxiety (Pearce et al., 2023).

Structural oppression, including barriers to stable employment, healthcare, and housing, worsens psychological distress by limiting recovery opportunities and perpetuating cycles of hardship (Gordon-Achebe et al., 2023; Nakphong et al., 2024). Economic factors, including income level and housing instability, showed a strong relationship with mental health outcomes. Our study suggests that individuals with lower incomes had higher odds of experiencing both episodic and chronic poor mental health compared to those with higher incomes. Additionally, individuals facing financial difficulties, such as employment loss or reduction, or challenges in paying utility bills, were more likely to experience chronic poor mental health. These findings align with those of Nakphong et al. (2024), who highlighted income inequality and housing instability as major contributors to psychological distress (Nakphong et al., 2024). Rapisarda et al. (2023) similarly found that economic hardship persisted as a significant stressor during and after the COVID-19 pandemic, reinforcing the long-term impact of financial insecurity on mental health (Rapisarda et al., 2023).

Access to healthcare and transportation barriers significantly influence mental health outcomes. Individuals who reported having trouble affording a doctor’s visit or a long period of time since a doctor’s visit generally reported worse mental health status. In addition, individuals facing transportation barriers had a higher likelihood of experiencing chronic poor mental health rather than episodic or no mental health issues. These findings are consistent with research by Cochran et al. (2022), which showed that structural barriers to healthcare access contribute to mental health disparities (Cochran et al., 2022). Additionally, this finding is supported by previous research studies, which suggest that transportation accessibility challenges are a critical determinant of mental health service utilization (Breslau et al., 2023; Smith et al., 2023).

All these findings underscore the importance of an integrated approach to handling various social determinants of mental health. Mental health disparities can be addressed through targeted policies and interventions that promote access to education, ensure food and housing stability, and mitigate economic hurdles (Brown et al., 2019; Gordon-Achebe et al., 2023; Rolfe et al., 2020). In addition, culturally sensitive interventions and improved access to mental health services, particularly for marginalized groups, are crucial for mitigating disparities (Kalibatseva & Leong, 2014; Molander et al., 2024; Newberry et al., 2024). Indeed, such multifactorial and tailored approaches to addressing SDoH can contribute to achieving sustainable development goals and build societal resilience (Oswald et al., 2024).

Based on our findings, future public health interventions should leverage intersectoral collaborations with the Health in All Policies (HiAP) approach for effective policies to routinely assess social risks. The HiAP approach will require public health to inform and educate stakeholders in other social sectors about the adverse mental health outcomes of policies insensitive to the negative impacts of social determinants, such as food insecurity, poor housing, transportation challenges, and financial hardship (G. H. Shah, 2021). Healthcare providers should build stronger partnerships with social service organizations to better meet patients’ unmet needs as part of a more comprehensive approach to mental health care. At the policy level, expanding access to affordable healthcare, strengthening food assistance programs, and investing in transportation and community resources could help alleviate chronic psychological distress among vulnerable populations. Addressing these broader social factors is crucial for advancing mental health equity.

This study has several limitations. First, the cross-sectional pattern of the BRFSS data restricts causal inference. Since the relationship between SDoH and mental health was analyzed using cross-sectional data, the relationships should be considered associations rather than assuming any causal relationships. Second, the study relies on self-reported days of poor mental health as measured by the BRFSS Survey, a broad and subjective indicator of emotional distress that does not equate to clinical diagnoses such as depression, anxiety, or other psychiatric disorders. Also, as the study depends on self-reported mental health data, which may be prone to recall and social desirability biases. Third, the current study might overlook certain confounding variables that could influence the observed relation, such as hereditary traits or access to mental health care. Fourth, while SNAP participation was included in the analysis, its frequency may not fully reflect the nuances of food security, particularly given that the sample was not limited to low-income individuals. Fifth, the analysis did not consider important factors like social support, loneliness, and a sense of belonging, all of which are known to influence mental health outcomes. The absence of these factors may limit a comprehensive understanding of the pathways through which SDoH impacts psychological well-being. Future studies should incorporate these psychosocial factors for a more comprehensive analysis. Sixth, the study does not explore potential variations across geographic regions (e.g., by state, urban vs. rural areas, or underserved regions), which could reveal important contextual disparities in both social determinants and mental health outcomes.

Regardless of its limitations, the study findings provide empirical evidence for healthcare providers and policymakers aiming to customize strategies and allocate resources efficiently. This research enhances nuanced understanding and explores deeper correlations between SDoH and the mental health status of U.S. adults that can curb prevalent mental health disparities to promote healthier societies moving forward. Furthermore, the current study makes significant contributions to the existing literature due to its strengths. It used a comprehensive, nationwide representative dataset (BRFSS) that ensures the generalizability of the results for the broader U.S. population. Furthermore, the multinomial logistic regression analysis provides a deeper insight into the association between multiple SDoH and varying levels of mental health outcomes.

Future research should employ longitudinal study designs to investigate the causal mechanisms between SDoH and mental health outcomes over time. Analyzing changes in SDoH, such as income variability or improvements in food security, can influence mental health trends. In addition, qualitative studies examining the lived experiences of individuals facing adverse social determinants of health (SDoH) can enhance quantitative findings. These types of studies provide a detailed understanding of the psychosocial pathways linking SDoH to mental health outcomes. Furthermore, future studies should consider integrating broader measures of SDoH, involving neighborhood conditions, discrimination, and social support networks to understand their combined impact on mental health. Lastly, intervention-based research is crucial for evaluating the effectiveness of policies and programs that focus on SDoH. For example, research investigating the impact of affordable housing initiatives or food assistance programs can contribute to the development of evidence-based policies. Future research studies should discuss the results and how they can be interpreted from the perspective of previous studies and of the working hypotheses. The findings and their implications should be discussed in the broadest context possible. Future research directions may also be highlighted.

## 5. Conclusions

Our study demonstrated the significant interaction between mental health and a range of SDoH, highlighting the critical importance of reducing socioeconomic gaps and improving SDoH that are likely crucial for promoting the mental health of the adult population. Policies focusing on education and transportation security, employment loss, equitable access to healthcare, and economic sustainability are crucial for minimizing psychological distress. Effective policy mobilization will require public health leadership to proactively form cross-sectoral collaborations through the HiAP approach. Policy- and practice-relevant future research evidence will be beneficial for informing interventions to mitigate these disparities, focusing on long-term relationships between SDoH and mental health status. Additionally, culturally appropriate interventions for marginalized groups and increased transportation accessibility could significantly increase mental health services utilization, ultimately improving mental health outcomes. Future research should examine causal pathways and the effectiveness of targeted interventions for addressing SDoH. Integrating qualitative methods could provide detailed insights to ensure that mental health strategies are both evidence-based and culturally resonant. Addressing the root causes of mental health disparities will enable policymakers to build resilience across communities and foster mental health equity.

## Figures and Tables

**Table 1:** Descriptive statistics of study respondent characteristics, 2023 BRFSS Survey.

Variable	Frequency	Percentage (%)
Gender		
Female	96,935	52.9
Male	86,383	47.1
Race/ethnicity		
Non-Hispanic White	135,289	73.8
Hispanic	17,048	9.3
Non-Hispanic Black	12,132	6.6
Other non-Hispanic	11,066	6
Multiracial non-Hispanic	7783	4.3
Age Groups		
Age >64	69,662	38.1
Age 55–64	32,997	18
Age 45–54	26,214	14.3
Age 35–44	23,831	13
Age 25–34	19,432	10.6
Age 18–24	11,182	6.1
Educational Level		
College 4 years or more	78,094	42.6
College 1–3 years	50,048	27.3
High school graduate/GED	44,363	24.2
Grade 9–11	6599	3.6
Elementary (grades 1–8)	3849	2.1
Never attended/kindergarten	365	0.2
Food Assistance Participation (SNAP) During the 12 Months Before the Survey		
Never	146,105	79.7
Rarely	15,214	8.3
Sometimes	13,382	7.3
Usually	3483	1.9
Always	5134	2.8
Annual Household Income (USD)		
200,000 or more	44,364	24.2
100,000 to 199,999	47,662	26
50,000 to 100,000	33,914	18.5
35,000 to <49,000	20,898	11.4
25,000 to <34,000	16,132	8.8
15,000 to 24,000	12,649	6.9
Less than 15,000	7699	4.2
Employment Loss/Reduction		
No	165,353	90.2
Yes	17,965	9.8
Unable to Pay Bills		
No	166,319	90.7
Yes	16,999	9.3
Transportation Barrier		
No	171,015	93.3
Yes	12,303	6.7
Marital Status		
Married	95,142	51.9
Never married	32,264	17.6
Divorced	23,098	12.6
Widowed	20,532	11.2
Member of unmarried couple	7699	4.2
Separated	4583	2.5
Transgender Status		
No	179,835	98.1
Yes, male to female	367	0.2
Yes, female to male	366	0.2
Yes, gender non-conforming	2750	1.5
Could Not Afford Doctor		
No	167,886	91.6
Yes	15,432	8.4
Time Since Last Checkup		
Within past year	147,728	80.6
Within past 2 years	15,766	8.6
Within past 5 years	8799	4.8
5 or more years	11,025	6
Home Ownership		
Own	130,012	70.9
Rent	42,712	23.3
Other arrangement	10,594	5.8

Abbreviations: SNAP, Supplemental Nutrition Assistance Program.

**Table 2:** Multinomial multivariable logistic regression analysis of poor mental health in a sample of 183,318 U.S. adults from the 2023 BRFSS Survey.

Demographic and SDoH Variables	(1–13) Days vs. 14+ Days of Poor Mental Health				0 Days vs. 14+ Days of Poor Mental Health 95% CI			
	AOR	95% CI		*p*	AOR	95% CI		*p*
		LL	UL			LL	UL	
**Gender**								
Female	(Ref)							
Male	**0.96**	0.94	0.99	<0.040	**0.92**	0.89	0.95	<0.001
**Race/Ethnicity**								
Hispanic	(Ref)							
Other non-Hispanic	1.02	0.85	1.23	0.802	1.01	0.8	1.23	0.926
Non-Hispanic Black	0.89	0.75	1.07	0.212	0.87	0.73	1.04	0.121
Non-Hispanic White	**0.73**	0.58	0.92	0.01	**0.73**	0.58	0.92	0.004
**Age group**								
Age > 64	(Ref)							
Age 55–64	**0.81**	0.8	0.83	<0.001	**0.5**	0.48	0.52	0.006
Age 45–54	**0.87**	0.85	0.89	<0.001	0.39	0.37	0.41	0.333
Age 35–44	**0.92**	0.9	0.94	<0.001	0.3	0.28	0.32	0.102
Age 25–34	**0.9**	0.87	0.92	<0.001	**0.21**	0.19	0.23	<0.001
Age 18–24	**0.83**	0.83	0.83	<0.001	**0.17**	0.15	0.19	<0.001
**Educational Level**								
≥4 years college	(Ref)							
Some college	**0.57**	0.34	0.96	0.033	0.66	0.39	1.12	0.123
High school graduate	0.59	0.35	1	0.05	0.44	0.44	1.39	0.401
Some high school	0.6	0.03	1.04	0.068	0.37	0.37	1.21	0.189
Elementary/never attended	0.66	0.3	1.47	0.309	0.17	0.17	1.03	0.057
**Food Assistance Participation** **(SNAP)**								
Never	(Ref)							
Rarely	1.46	0.99	2.17	0.058	0.89	0.68	1.17	0.602
Sometimes	1.33	0.99	1.97	0.155	0.86	0.68	1.1	0.758
Usually	**1.75**	1.23	2.51	0.002	1.04	0.8	1.37	0.227
Always	**1.7**	1.2	2.4	0.003	0.86	0.49	1.52	0.399
**Utility Bill Payment**								
No	(Ref)							
Yes	**0.47**	0.19	0.97	0.031	**0.24**	0.09	0.6	0.003
**Annual Household Income (USD)**								
200,000 or more	(Ref)							
100,000 to 199,000	**1.36**	1.27	1.45	<0.001	**0.45**	0.34	0.6	<0.001
50,000 to 100,000	**1.46**	1.46	1.54	<0.001	**0.57**	0.48	0.69	<0.001
35,000 to < 49,000	**1.33**	1.33	1.4	<0.001	**0.78**	0.67	0.91	0.002
25,000 to 34,000	**1.26**	1.26	1.34	<0.001	**0.81**	0.69	0.95	0.011
15,000 to 24,000	**1.17**	1.17	1.27	<0.001	0.86	0.71	1.04	0.118
Less than 15,000	1.03	1.03	1.14	0.572	0.83	0.64	1.06	0.129
**Employment Loss/Reduction**								
No	(Ref)							
Yes	1.17	0.9	1.51	0.236	**0.84**	0.77	0.91	<0.001
**Transportation barrier**								
No	(Ref)							
Yes	**0.35**	0.14	0.88	0.026	**0.21**	0.07	0.58	0.003
**Marital Status**								
Married	(Ref)							
Divorced	0.93	0.63	1.37	0.697	0.76	0.53	1.09	0.134
Widow	**0.69**	0.47	0.99	0.043	**0.68**	0.47	0.96	0.031
Separated	0.74	0.49	1.11	0.147	0.91	0.6	1.4	0.679
Never married	0.74	0.52	1.06	0.1	1.1	0.76	1.6	0.606
**Transgender Status**								
No	(Ref)							
Yes, male to female	1.23	0.64	2.38	0.535	1.34	0.69	2.6	0.386
Yes, female to male	**2.56**	1.11	5.93	0.028	2.05	0.89	4.74	0.092
Yes, gender non-conforming	**2.6**	1.24	5.46	0.011	**2.34**	1.12	4.89	0.023
**Could not afford a doctor**								
No	(Ref)							
Yes	0.59	0.29	1.195	0.142	**0.37**	0.21	0.65	0.004
**Length of time since last check-up**								
5+ years	(Ref)							
Within past 5 years	**2.98**	1.57	5.67	<.001	**2.028**	1.275	3.225	0.003
Within past 2 years	**2.5**	1.32	4.75	0.005	1.91	1.202	3.033	0.006
Within past years	**2.16**	1.14	4.1	0.018	**1.73**	1.09	2.75	0.019
**Homeownership**								
Own	(Ref)							
Rent	**1.34**	1.03	1.72	0.026	0.9	0.76	1.08	0.266
Other arrangement	**1.50**	1.08	2.00	0.022	0.95	0.74	1.20	0.250

Abbreviations: AOR, adjusted odds ratio; CI, confidence interval; LL, lower limit; UL, upper limit; Ref, reference category; SNAP, Supplemental Nutrition Assistance Program. Note: Bolded AORs indicate that adjusted odds of the outcome variable are significantly different at *p* ≤ 0.05 for an attribute of a variable compared to the reference category.

## Data Availability

Data can be accessed on 16 September 2024, at https://www.cdc.gov/brfss/annual_data/annual_2023.html.
